# Epithelial Proinflammatory Response and Curcumin-Mediated Protection from Staphylococcal Toxic Shock Syndrome Toxin-1

**DOI:** 10.1371/journal.pone.0032813

**Published:** 2012-03-14

**Authors:** Matthew M. Schaefers, Laura M. Breshears, Michele J. Anderson, Ying-Chi Lin, Alex E. Grill, Jayanth Panyam, Peter J. Southern, Patrick M. Schlievert, Marnie L. Peterson

**Affiliations:** 1 Department of Experimental and Clinical Pharmacology, University of Minnesota, Minneapolis, Minnesota, United States of America; 2 Department of Pharmaceutics, University of Minnesota, Minneapolis, Minnesota, United States of America; 3 Department of Microbiology, University of Minnesota, Minneapolis, Minnesota, United States of America; 4 Department of Microbiology, University of Iowa, Iowa City, Iowa, United States of America; 5 School of Pharmacy, Kaohsiung Medical University, Kaohsiung, Taiwan; The University of Hong Kong, Hong Kong

## Abstract

*Staphylococcus aureus* initiates infections and produces virulence factors, including superantigens (SAgs), at mucosal surfaces. The SAg, Toxic Shock Syndrome Toxin-1 (TSST-1) induces cytokine secretion from epithelial cells, antigen presenting cells (APCs) and T lymphocytes, and causes toxic shock syndrome (TSS). This study investigated the mechanism of TSST-1-induced secretion of proinflammatory cytokines from human vaginal epithelial cells (HVECs) and determined if curcumin, an anti-inflammatory agent, could reduce TSST-1-mediated pathology in a rabbit vaginal model of TSS. TSST-1 caused a significant increase in NF-κB-dependent transcription in HVECs that was associated with increased expression of TNF- α, MIP-3α, IL-6 and IL-8. Curcumin, an antagonist of NF-κB-dependent transcription, inhibited IL-8 production from *ex vivo* porcine vaginal explants at nontoxic doses. In a rabbit model of TSS, co-administration of curcumin with TSST-1 intravaginally reduced lethality by 60% relative to 100% lethality in rabbits receiving TSST-1 alone. In addition, TNF-α was undetectable from serum or vaginal tissue of curcumin treated rabbits that survived. These data suggest that the inflammatory response induced at the mucosal surface by TSST-1 is NF-κB dependent. In addition, the ability of curcumin to prevent TSS *in vivo* by co-administration with TSST-1 intravaginally suggests that the vaginal mucosal proinflammatory response to TSST-1 is important in the progression of mTSS.

## Introduction

*Staphylococcus aureus* is a significant human pathogen that causes a wide range of diseases, including menstrual toxic shock syndrome (mTSS). Staphylococcal mTSS is an acute-onset, potentially fatal, multi-system illness characterized by fever, hypotension, sunburn-like rash, peeling of the skin upon recovery, and multi-organ dysfunction [1]–[3]. The staphylococcal superantigen (SAg), Toxic Shock Syndrome Toxin-1 (TSST-1), causes the majority of mTSS cases by penetrating the vaginal mucosa and non-specifically cross-linking Major Histocompatibility Complex (MHC) class II molecules with T-cell receptors (TCR) causing massive systemic cytokine release from T cells (IL-2, TNF-β, and IFN-γ) and macrophages (IL-1β and TNF-α). However, the initial effects of *S. aureus* TSST-1 on the vaginal mucosa, and the outcomes of these initial effects, which lead to mTSS are unclear and are the focus of this study.

SAgs, including TSST-1, induce changes in cellular morphology and secretion of proinflammatory cytokines/chemokines from vaginal, bronchial, nasal, and intestinal epithelial cells [4]–[7]. The proinflammatory effect of TSST-1 on vaginal epithelial cells may be critical to mTSS progression as glycerol monolaurate (GML), a non-specific anti-inflammatory agent, protected against TSS in a rabbit vaginal model [8], [9]. However, the vaginal epithelial cell proinflammatory signaling pathways activated in response to TSST-1 are unknown.

Staphylococcal Enterotoxin (SE)C 1, another SAg, induces cytokine production via an NF-κB-dependent mechanism in human peripheral blood mononuclear cells (PBMCs) [10]. NF-κB regulates the expression of many genes, including growth factors, cytokines, chemokines, and cell adhesion molecules in response to external stimuli [11]–[13]. Many of the vaginal epithelial genes that are upregulated in response to TSST-1 are known NF-κB-regulated genes [11]. We hypothesized the NF-κB pathway was critical for the epithelial proinflammatory response to TSST-1. Blocking this pathway and inhibiting the production of epithelial cytokines would prevent progression to mTSS [4], [5], [7].

Curcumin, a component of the Indian spice Turmeric (*Curcuma longa*), was demonstrated to be effective in reducing the inflammatory response and negative consequences of bacterial infections *in vitro* and *in vivo* [14]–[21]. The mechanism by which curcumin mediates these effects is thought to be via inhibition of multiple proinflammatory pathways including NF-κB.

We investigated the effects of TSST-1 on immortalized human vaginal epithelial cells (HVECs). Our findings demonstrate that TSST-1 causes an increase in NF-κB -dependent reporter transcription in HVECs suggesting that NF-κB is a component of the TSST-1-induced signaling pathway. We further explored the ability of curcumin, to prevent TSS in a rabbit model of mTSS [15], [22]. Intravaginal co-administered curcumin and TSST-1 prevents TSS in a rabbit model potentiated with LPS. Together, these data suggest that the inflammatory response induced at the mucosal epithelia by TSST-1 is a critical step in the progression of TSS. Our curcumin data indicate a role for compounds that inhibit local mucosal epithelial cytokine and chemokine production in the prevention of SAg-related diseases.

## Results

A previous study reported an increase in the production of cytokines/chemokines by HVECs in response to TSST-1, including CCL20 (MIP-3α), CXCL1, CXCL2, CXCL3, IL-1β, IL-6, IL-8, and TNF-α [4]. We hypothesized that vaginal mucosal proinflammatory cytokines contribute to TSS by an outside-in proinflammatory signaling mechanism, which ultimately disrupts the mucosa and allows TSST-1 to penetrate into the submucosa and bloodstream.

### TSST-1 induces cytokines and chemokines and activates the NF-κB pathway

**Figure 1:**
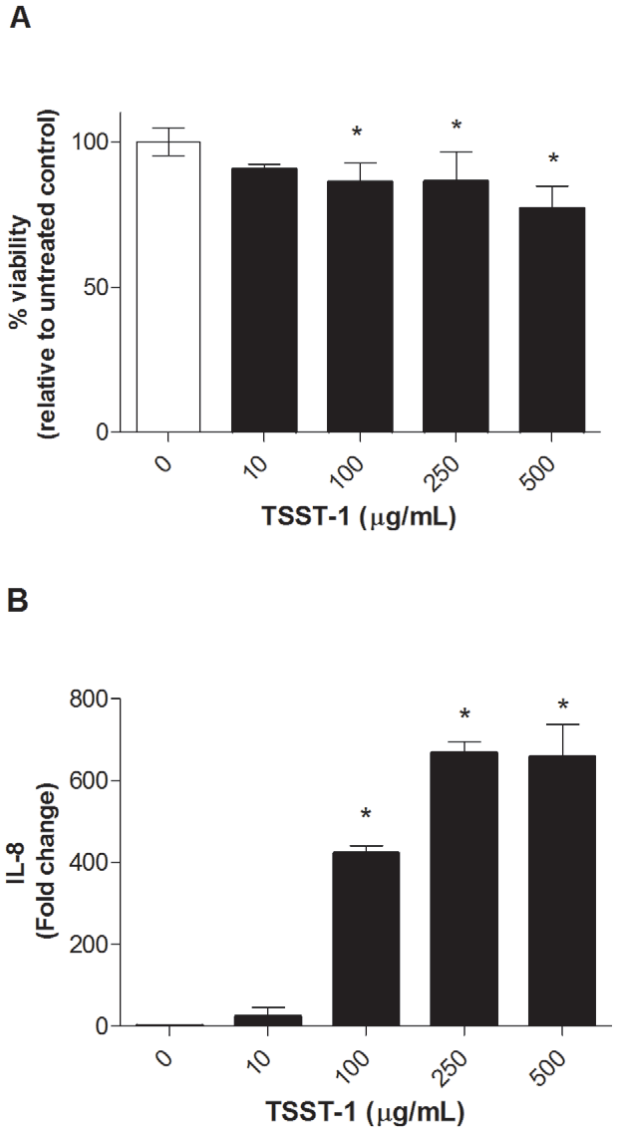
TSST-1 induces IL-8 and activates NF-κB from HVEC with minimal toxicity. HVEC were exposed to TSST-1 (10–500 µg/ml) for 6 h, then cell viability (A) and IL-8 production (B) were determined. Viability was determined using an MTT assay. IL-8 was measured from culture supernatants by ELISA and expressed as fold increase in IL-8 production compared to media only control (TSST-1 0 µg/ml). Data presented are representative of three independent experiments done in triplicate, mean ± SD. * denotes p<0.05 compared to media only control.

Previous studies were expanded using an unrelated, immortalized HVEC line constructed from another woman, which was obtained from American Type Culture Collection (ATCC) [23]. HVECs were exposed to increasing concentrations of TSST-1 (10–500 µg/ml) for 6 h, whereupon HVEC viability or secreted IL-8 were determined (Fig. 1 A and B). TSST-1 had minimal effect on HVEC viability with ≥75% of HVECs remaining viable following 6 h exposures of 10 to 500 µg/ml. IL-8 secretion by HVECs maximized at TSST-1 250 µg/ml, with an estimated half maximal effective concentration (EC50) of 100 µg/ml.

**Figure 2:**
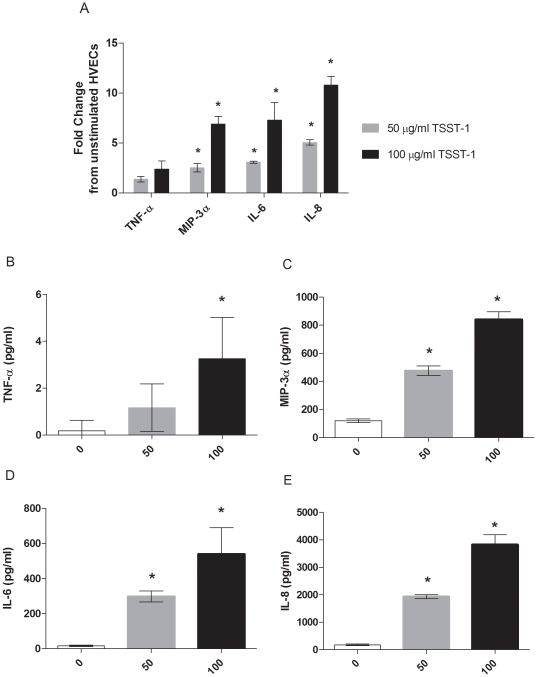
TSST-1 induces cytokines from immortalized HVEC. HVECs were exposed to TSST-1 (50 and 100 µg/ml) or media only controls (0 µg/ml) for 6 h, then RNA was harvested and supernates collected. (A) changes in expression of proinflammatory genes were measured by real time RT-PCR, and (B–E) secreted cytokines were measured via multiplex cytokine array. Data presented in 2A are representative of three independent experiments conducted in triplicate, data presented in 2B–E are from one experiment conducted in triplicate, mean ± SD. * denotes p<0.05 compared to media only control.

Confluent HVECs exposed to TSST-1 (50 or 100 µg/ml) for 6 h produced multiple proinflammatory cytokines/chemokines. Changes in gene transcription were measured by collecting total cellular RNA and conducting real time RT-PCR. Transcription of cytokines and chemokines was upregulated 1.5 to 12 fold over 6 h in response to TSST-1 compared to media controls (Fig. 2 A). Multiplex cytokine array of HVEC supernates measured IL-6, IL-8, MIP-3α, and TNF-α, which were significantly upregulated in a dose dependent manner in response to TSST-1 (Fig. 2 B–E). Transcription and protein expression correlated, as IL-6, IL-8, MIP-3α, and TNF-α were upregulated in both assays.

**Figure 3:**
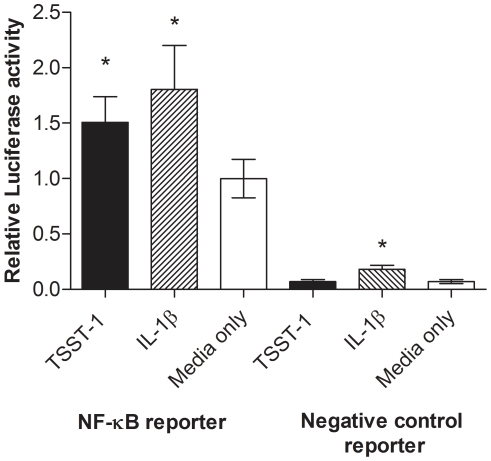
TSST-1 activates NF-κB in HVEC. HVECs were transfected with plasmids containing NF-κB response elements or a basal promoter (negative control), which control expression of luciferase. Transfected cells were then treated with TSST-1 (250 µg/ml) or left untreated for 4 h, then luciferase activity was measured. Data presented are from 15 replicates obtained from three independent experiments, mean ± SD. * denotes p<0.001 compared to corresponding media only control.

Since HVEC cytokines were upregulated in response to TSST-1, the NF-κB pathway was investigated as a possible downstream effector of TSST-1 signaling. NF-κB-responsive luciferase reporters measured NF-κB activation in response to TSST-1 or IL-1β (positive control) [24]. TSST-1 induced a significant increase of >50% in NF-κB–dependent transcription compared to media alone at 4 h (Fig. 3). IL-1β induced a significant increase of 80% in NF-κB activity compared to media only. No luciferase activation was detected in cells transfected with a negative control plasmid with a minimal promoter, indicating the observed luciferase activity was NF-κB-dependent. There was a significant increase in NF-κB activity in HVECs transfected with the negative control reporter when treated with IL-1β. This activation was attributed to IL-1β activating the minimal promoter that the negative control reporter contains. This activation was small and not seen when stimulated with TSST-1. These data are consistent with NF-κB activation by HVECs in response to TSST-1.

### Curcumin is non-toxic to vaginal mucosa and inhibits IL-8 production

**Figure 4:**
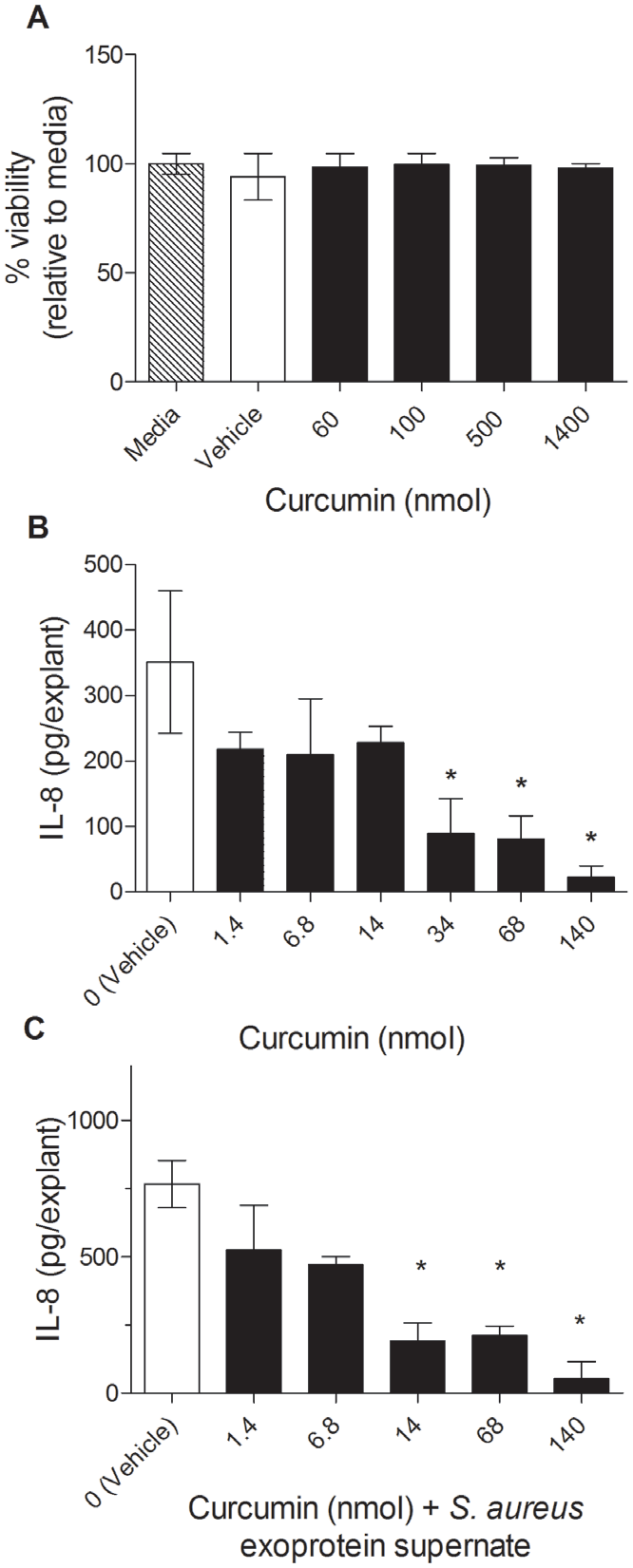
Curcumin inhibits *S. aureus* exoprotein-induced IL-8. (A) Porcine vaginal explants (5 mm) were treated with curcumin in 5 µl of 100% DMSO and incubated for 18 h. Tissue viability was measured using a MTT assay and normalized to tissue left untreated (media only: no DMSO or curcumin). * denotes p<0.05 compared to vehicle control (0 nmol curcumin in 100% DMSO) by ANOVA. Curcumin inhibits IL-8 production. Porcine vaginal explants were left unstimulated (B) or stimulated with filtered *S. aureus* culture supernates for 1 h (C) and then exposed to curcumin (5 µl/explant in 10% DMSO) and incubated for an additional 6 h. Tissue was disrupted and IL-8 was measured by ELISA. * denotes p<0.05 compared to vehicle control (0 nmol curcumin in 10% DMSO) by ANOVA. Data presented are representative of three independent experiments done in triplicate, mean ± SD.

Because TSST-1 activates and NF-κB reporter and induces NF-κB –regulated cytokines in HVECs, curcumin, a known NF-κB inhibitor, was evaluated for its potential to reduce cytokine production and prevent TSS. A full thickness *ex vivo* porcine vaginal mucosal model was used to evaluate curcumin's toxicity and potential to reduce proinflammatory cytokine production. Porcine mucosa provides an excellent tissue model for *in vitro* correlates of the effects of infectious and chemical entities on human tissue [25]. Tissue explants were exposed to curcumin (60–1350 nmol/explant) for 18 h and cell viability was measured using an MTT assay. No reduction in viability at any dose was noted when compared to controls (Fig. 4 A). Curcumin was tested for its ability to inhibit staphylococcal exoprotein-induced IL-8 in the *ex vivo* porcine vaginal mucosa. Porcine vaginal mucosal explants were exposed for 1 h to filtered *S. aureus* (MNPE) overnight supernates, which contained SAgs (TSST-1 and SEC), cytolysins, and proteases for maximal mucosal stimulation. Curcumin was then applied and explants were incubated for an additional 6 h (Fig. 4 B). Curcumin inhibited IL-8 production in response to *S. aureus* exoproteins with doses as low as 14 nmoles of curcumin per tissue explant. The IC50 in this experiment was estimated to be 10 nmol. Higher doses of curcumin resulted in additional decreases in IL-8 levels below those of untreated controls.

### Curcumin protects rabbits in a lethal vaginal TSS model

**Figure 5:**
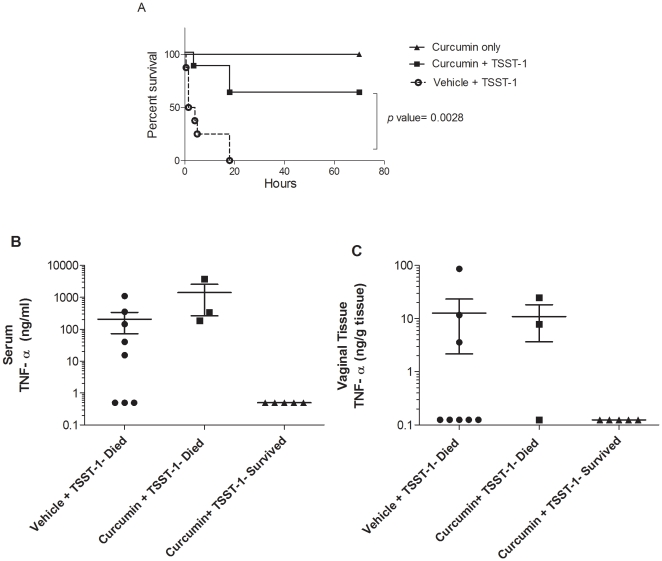
Curcumin reduces TSST-1-induced death in a rabbit model of TSS. (A) Curcumin protects rabbit from lethal TSS. TSST-1 (10 µg) in 3% DMSO was administered intravaginally with or without curcumin (1.62 µmol) (n = 8 rabbits per group). Four hours later LPS (0.1 µg/kg) was administered via ear marginal vein and animals monitored for survival. Curcumin significantly reduced TSS-induced mortality compared to untreated (p<.0028, Log-rank test). Four rabbits (given curcumin only, triangles) displayed no signs of disease and survived the entirety of the experiment. Curcumin decreases TNF-α in serum (B) and vaginal tissue (C) from surviving rabbits in a lethal model of TSS. TNF-α in serum and homogenized vaginal tissue was determined using ELISA. Each data point corresponds to data from 1 rabbit measured in duplicate. Data presented as mean ± SEM.

A previously described rabbit vaginal model of TSS was utilized to determine if curcumin applied vaginally could prevent TSST-1-induced mortality [26]–[28]. TSST-1 was administered vaginally via a catheter with or without curcumin (1.62 µmol) (8 rabbits per group). Curcumin protected 5 of the 8 rabbits from TSS and death over approximately 3 days compared to 0 of 8 positive control animals (Fig. 5 A). This reduction in mortality was significant (*p* value<0.0028, Log-rank test). Four rabbits given curcumin only (no TSST-1 or LPS) displayed no signs or symptoms of disease and survived the entirety of the experiment.

At the time of death or when found dead the next morning, serum and vaginal tissue were harvested to determine levels of TNF-α, as this is a principal cytokine that mediates hypotension in TSS and causes death [29]. TNF-α in the serum was detected in 8 of 11 rabbits that died during the experiment, while none of the rabbits that survived had detectable serum TNF-α (Fig. 5 B). TNF-α was detectable in the vaginal tissue in 5 of 11 rabbits that died during the experiment (all 5 had detectable amounts of serum TNF-α) (Fig. 5 C). Rabbits that survived did not have detectable vaginal TNF-α. Curcumin concentrations were quantified to determine curcumin persistence in the vaginal tissue after intravaginal administration. Seven of 8 treated rabbits had detectable curcumin in the vaginal tissue at the time of death or completion of the study. At 70 h post curcumin application, 0.8% of the original dose remained. Curcumin was undetectable in all serum and liver tissue samples (data not shown).

## Discussion

The importance of a vaginal mucosal outside-in proinflammatory signaling mechanism has been suggested to be critical to disease progression for staphylococcal SAg-mediated TSS [30]. This mechanism is similar to the vaginal transmission of simian immunodeficiency virus (SIV) in a rhesus macaque (*Macacca mulatta*) model [31]–[33]. In these diseases, toxins or antigens activate vaginal epithelial cells to produce proinflammatory cytokines/chemokines, which disrupt the epithelium and attract neutrophils, APCs, and T lymphocytes. This immune cell migration in turn provides the necessary host cell targets to cause and perpetuate disease in the presence of either SAgs or SIV. HVECs exhibit a proinflammatory response to TSST-1 including the increased production of cytokines/chemokines [4].

In a previous study, we demonstrated that the initial localized mucosal inflammatory response to TSST-1 is critical for TSS progression [4]. The proinflammatory response and progression to TSS are abrogated by a mutation in a HVEC proinflammatory residue of TSST-1 (D130A) [33]. This residue is located in a 12-amino acid region, which is separate from the MHC class II molecule and TCR binding domains, and is critical for the induction of proinflammatory cytokines from HVECs [33], [34]. TSST-1 D130A is unable to induce cytokines from HVECs and is non-lethal when administered vaginally in the same rabbit TSS model used in the current study. However, this mutant toxin maintains its ability to cause disease when administered systemically [33]. These data indicate that local mucosal effects are critical for TSST-1 to cause systemic disease and underscore the need for an in-depth analysis of the epithelial response to TSST-1 and other SAgs in order to appreciate the role the epithelium plays in the initiation of disease and how we might better prevent these diseases.

The mechanisms of SAg stimulation of cytokines from epithelial cells are not well characterized. In this study, we observed a proinflammatory response induced by TSST-1 from an immortalized HVEC line obtained from ATCC and determined that the transcription factor NF-κB is activated in HVECs in response to TSST-1. The activation of NF-κB is likely responsible for the observed cytokine production as many of the TSST-1-induced cytokines are known to be regulated by NF-κB [11]. These cytokines/chemokines are responsible for migration of immune cells to the vaginal mucosa [35], [36]. Progression of mTSS occurs as these immune cells are activated in a non-antigen specific manner by the superantigenic property of TSST-1. The receptor(s) and signaling pathway(s) responsible for the TSST-1-induced cytokine response in HVECs remain poorly characterized and are currently under investigation.

The importance of the inflammatory response at mucosal interfaces suggests that local anti-inflammatory compounds may be of use in the prevention of disease. Curcumin is a component of the Indian spice Turmeric (*C. longa*) that has anti-inflammatory, anti-angiogenesis, and antimicrobial properties [14]–[16]. Curcumin was chosen as a potential anti-TSS agent because it is known to block cytokine-mediated NF-κB activation and proinflammatory gene expression by inhibiting inhibitory factor I-κB kinase activity [15], [22], [37]. In addition, the proinflammatory effects of SAgs on immune cells, such as SEA- and SEB-induced cytokine production from PBMCs and SE-induced fever in rabbits, are inhibited by curcumin [38].

Inhibition of IL-8 production by curcumin from *ex vivo* porcine vaginal explants indicates that curcumin is anti-inflammatory at the mucosal surface. The doses effective in reducing IL-8 were far lower than doses that are toxic to the tissue, suggesting a wide therapeutic window. Curcumin significantly decreased TSST-1-induced lethality in rabbits by 60% suggesting that inhibition of inflammation at the mucosal surface has therapeutic potential. TNF-α, a major mediator of TSS [1]–[3], was undetectable from serum or vaginal tissue of surviving curcumin-treated rabbits.

The dose of curcumin used in the rabbit model (530 nmol/cm^2^) was higher than the minimum effective dose in the full thickness porcine tissue model (34 nmol or 420 nmol/cm^2^) but lower than the highest dose tested in the porcine tissue toxicity experiment (1350 nmol or 17,000 nmol/cm^2^). In other words, the *in vivo* rabbit experiment was performed using a concentration of curcumin within the therapeutic range identified using the porcine *ex vivo* model. Therefore, we believe that curcumin concentrations in this range are safe for topical vaginal application.

Curcumin has previously been shown to reduce inflammation. For example, orally administered curcumin inhibited *Klebsiella pneumoniae*-induced neutrophil infiltration into lung tissue and local TNF-α production and diminished the production of proinflammatory proteins in a murine lung model of infection [20]. This reduction in inflammation and related cytokines occurs without reduction in bacterial load indicating that the reduction in inflammation is a result of curcumin's anti-inflammatory activity not any anti-microbial properties. These data in addition to our current findings suggest that curcumin's ability to inhibit the proinflammatory effects of an infectious or toxigenic agent on a mucosal surface may reduce or prevent clinical disease.

The rabbit vaginal TSS model is not without limitations, including the LPS enhancement of TSST-1, which causes 100% mortality in the animals by 24 h. This clearly allows for delineation of curcumin effectiveness (survival at 24 h); however, since many of the animals died within 6 to 12 h of TSST-1 exposures, the short exposure time limited our ability to observe significant histological changes in the mucosa, including the infiltration of immune cells due to exposure to TSST-1.

The findings presented here demonstrate the importance of local inflammation caused by superantigens in the progression to TSS. Additionally, curcumin was identified as a potential compound for the prevention of mTSS. Based on similarities in the proinflammatory response induced by other SAgs from epithelial cells derived from multiple tissues [4]–[7], we predict that curcumin will inhibit the proinflammatory response in these models as well. Curcumin may thus have the potential to prevent other SAg-mediated diseases such as atopic dermatitis, staphylococcal food-borne disease, and non-menstrual TSS [39], all of which initiate as a local proinflammatory response. We hypothesize that administration of topical anti-inflammatory agents such as curcumin would prevent this proinflammatory response, thereby preventing progression to clinical symptoms.

## Materials and Methods

### Toxin purification and quantification

TSST-1 was purified by an established method using successive thin layer isoelectric focusing [40], [41]. TSST-1 (1 mg/ml), thus prepared, contained no detectable LPS, as tested by *Limulus* assay (Sigma, St. Louis, Mo.) nor were peptidoglycan, lipoteichoic acid, hemolysin, protease, or lipase (<1 part per 10^6^) detectable by combinations of bioassay and SDS-PAGE. Comparable results were obtained with multiple batches of TSST-1.

### Human vaginal epithelial cells

HVECs were purchased from ATCC (Manassas, VA) (CRL 2616) and have been previously described [42]. This cell line was derived from normal vaginal mucosal tissue taken from a premenopausal woman and transformed with the E6/E7 genes of human papilloma virus 16. HVECs were cultured in keratinocyte serum-free medium (KSFM) (Gibco, Invitrogen, Carlsbad, CA) on plastic at 37°C in 7% CO_2_, and supplemented with bovine pituitary extract and epidermal growth factor (as provided by the manufacturer), calcium chloride (0.4 mM), penicillin (50 IU/ml), streptomycin (50 µg/ml), and amphotericin B (2.5 µg/ml) (Fungizone; Gibco, Invitrogen).

### Cytokine assays

HVECs were grown to confluence in six well plates and then exposed to purified TSST-1 (10–500 µg/ml) for 6 h in KSFM without antibiotics. Control wells of HVECs contained KSFM without antibiotics and without TSST-1. For some experiments IL-8 was measured as a marker cytokine; cell culture supernates were collected and tested for IL-8 by ELISA (R & D Systems, Minneapolis, MN) according to manufacturer directions. Unless otherwise specified, all experiments were conducted in triplicate, and graphs represent means ± standard deviation (SD).

In other experiments, supernates were removed and saved for measuring secreted cytokines using the Luminex (Austin, TX) multiplex assay, and human specific Fluorokine MAP multiplex assay kits (R & D Systems, Minneapolis, MN). Lower limits of detection: TNF-α 1.0 pg/ml, IL-8 & IL-6 0.5 pg/ml, and MIP-3α 0.47 pg/ml. Total RNA was isolated using RNeasy Mini kit (Qiagen, Valencia, CA), and cDNA was generated using reverse transcription (iScript, Bio-Rad Corporation) following the manufacturer's instructions. Expression of cytokine genes was measured using real time RT-PCR with SYBR® Advantage® Premix (Clonetech, Mountain View, CA) on MYiQ™ iCycler (Bio-Rad Corporation). Expression of cytokine genes was normalized using expression of the human hypoxanthine phosphoribosyltransferase (HPRT) gene in untreated samples as a control with use of primers listed in Table 1 (the ΔΔ^CT^ method).

**Table 1:** Primers used in reverse transcription polymerase chain reaction.

Gene	Primer Sequence
IL-8	Forward- 5′ AGCCTTCCTGATTTCTGCAGCTCTReverse- 5′ AATTTCTGTGTTGGCGCAGTGTGG
IL-6	Forward- 5′ CATGTGTGAAAGCAGCAAGAGGCReverse- 5′ CACCAGGCAAGTCTCCTCATTGAA
TNF-α	Forward- 5′ TGCCTGCTGCACTTTGGAGTGATReverse- 5′ GGTTCGAGAAGATGATCTGACTGCCT
MIP-3α	Forward- 5′ TTGCTCCTGGCTGCTTTGATGTReverse- 5′ TGCCGTGTGAAGCCCACAATAA
HPRT	Forward- 5′ GGTGAAAAGGACCCCACGAAReverse- 5′ AGTCAAGGGCATATCCTACA

### Cell viability assay

Cell survival was measured per manufacturer instructions after 6 h exposure to TSST-1 using CellTiter 96®Aqueous One Solution Cell Proliferation Assay (Promega, Madison, WI).

### NF-κB activation assay

HVECs were seeded in KSFM with antibiotics and growth factors into 96-well plates at 15,000 cells/well. After 24 h, cells were transfected with NF-κB luciferase reporter, negative control, or positive control plasmids (SA Biosciences, Frederick, MD) using the Genejuice transfection reagent (EMD4 Biosciences, Gibbstown, NJ). After 48 h, the cells were stimulated with 50 ng/ml IL-1β (Biolegend), 250 µg/ml TSST-1, or were left unstimulated. After 4 h, the level of NF-κB activation was determined using the Dual-Glo luciferase assay (Promega, Madison, WI) and a Beckman Coulter LD400 luminometer.

### *Ex vivo* porcine vaginal mucosa culture

Specimens of normal porcine vaginal mucosa were excised from animals at slaughter and transported to the laboratory to be processed as previously described [43]. The explants were then placed mucosal side up on a PET track-etched 0.4 µm cell culture insert (BD Bioscience, Franklin Lakes, NJ) in 6-well plates containing fresh serum and antibiotic-free RPMI 1640. The mucosal surface was continually exposed to air.

### Evaluation of curcumin cytotoxicity

Viability of the *ex vivo* porcine vaginal tissue explants was quantified using Cell Growth Determination kit (Sigma, St. Louis, MO). . Explants were incubated with various amounts of curcumin (Sigma, St. Louis, MO) in 100% DMSO at 37°C, 7% CO_2_ for 24 h. The effect of each formulation on tissue viability was expressed relative to untreated tissue control. SDS was used as a positive control for cytotoxicity (data not shown) [43].

### Inhibition of IL-8 from *ex vivo* porcine vaginal tissue by curcumin

Porcine vaginal explants (5 mm), (n = 3 per treatment)were exposed to curcumin (1.35–135 nmoles/explant) in 10% DMSO for 6 h. In some experiments, tissue explants were exposed to filtered (0.2 µm filter size) overnight *S. aureus* MNPE culture supernates [containing SEC, TSST-1, and α-toxin [44], and no live bacteria] for 1 h. Curcumin (13–135 nmoles/explant) in 10% DMSO was then added to the explants. Following an additional 6 h incubation tissue explants were placed into 0.25 ml PBS and vortexed at maximum speed for 2 min. This solution was clarified by centrifugation (14,000 g for 2 min), and supernate was removed and saved for IL-8 measurement via porcine specific ELISA (R & D Systems, Minneapolis, MN) following manufacturer's protocol.

### Rabbit model of mTSS

#### Ethics statement

All animal studies were performed in accordance with requirements established by the University of Minnesota Institutional Animal Care and Use Committee (IACUC), with specific approval given for this work under protocol 0908A71722 (project name Staphylococcal Superantigen Interactions With Vaginal Epithelium). A rabbit vaginal mTSS model was used to determine the ability of curcumin to inhibit TSST-1 induced proinflammatory effects on vaginal mucosal with subsequent TSS and lethality in rabbits, which was described elsewhere [26]–[28]. This model utilizes the ability of superantigens to enhance shock caused by LPS from *Salmonella enterica* serovar Typhimurium and causes mortality due to TSS within 24 h. Rabbits were anesthetized with ketamine (25 mg/kg; Phoenix Pharmaceuticals, Inc., St. Joseph, MO) and xylazine (20 mg/kg; Phoenix Pharmaceuticals, Inc.) prior to insertion of catheters into rabbit vaginas. TSST-1 (10 µg) was administered vaginally in 0.2 ml in 3% DMSO with or without curcumin (1.62 µmoles). LPS (0.1 µg/kg) was administered intravenously in the marginal ear veins 4 h after instillation of TSST-1. Additionally, 4 rabbits were given curcumin (1.62 µmoles) alone to determine the effect of curcumin alone on the rabbits. Rabbits were monitored for an additional 66 h. Rabbits displaying signs of severe illness (failure to right themselves and exhibit escape behavior), or those remaining healthy at the conclusion of the experiment, were euthanized with Beuthanasia D (1 ml/kg, Schering-Plough Animal Health Corp., Union, NJ) according to the University of Minnesota Institutional Animal Care and Use Committee requirement. Post mortem blood was collected via cardiac puncture and allowed to clot in serum collection tubes (BD Vacutainer, Franklin Lakes, NJ), and serum was separated by centrifugation (1,000 g for 10 min) and stored at −20°C. Vaginal and liver tissues were removed and stored at −20°C.

### TNF-α determination in rabbit serum and vaginal tissue

Vaginal tissue was placed in 0.25 ml PBS with 1% BSA and 0.05% Tween 20 and homogenized (three 20 s bursts at maximum power). Homogenates were clarified by centrifugation (14,000 g for 2 min). The clarified homogenates were stored at −20°C until analysis. TNF-α was measured in serum and vaginal tissue using rabbit specific ELISA (BD Bioscience, Franklin Lakes, NJ) following the manufacturer's protocol. For serum samples, the lower limit of detection was 0.5 ng/ml and 0.125 ng/tissue section in vaginal tissue.

### Curcumin determination

Curcumin concentration in tissue was analyzed using a previously established liquid chromatography-mass spectrometry (LC-MS) assay [45]. Tissues were homogenized and lyophilized. Curcumin was extracted from dry tissue using ethyl acetate (2 mL) overnight. Extracts were evaporated under nitrogen stream and then reconstituted in methanol. Chromatography was performed on an Agilent Technologies (1200 series) system with negative ESI connected to a TSQ Quantum system. An Agilent XDB-C18 1.8 µm, 4.6×50 mm column was used for separation. Mobile phase consisted of (A) 10 mM ammonium acetate and (B) acetonitrile. Gradient flow (0.5 mL/min) with a total run time of 10 min was used: 0–4.5 min: 45–100% B, 4.5–5.5 min: 100% B, 5.5–6 min: 100-45% B, 6–10 min: 45% B. The following mass transitions were monitored: curcumin - 367→216, hydroxybenzophenone 197→92. The lower limit of quantification was 0.15 nmoles/tissue section or 0.15 nmoles/ml serum.To estimate the total amount of curcumin within the tissue at late (70 h post curcumin dose) time point, the following calculation was made: Average curcumin concentration (nmoles/mg dry tissue weight)×9.1 mg tissue weight (average tissue section weight)×4 (approximately 25% of total vaginal tissue was used for curcumin determination) = estimated total vaginal curcumin.

### Statistical analysis

Unless otherwise specified, significance was determined by one way ANOVA using Dunnett's multiple comparison test, comparing experimental columns to control column (GraphPad Prism, La Jolla, CA). Significance between curcumin- and untreated mortality in rabbits was determined by Log-rank test.
